# Supplementary material for: Bardet-Biedl syndrome proteins modulate the release of bioactive extracellular vesicles
Source: Nat Commun. 2021 Sep 27;12:5671. doi: 10.1038/s41467-021-25929-1 (PMC8476602; doi:10.1038/s41467-021-25929-1)
Supplement: Supplementary file 1 — Supplementary Information [file 41467_2021_25929_MOESM1_ESM.pdf]

Supplementary Information

Supplementary Methods

Cell Survival Assay

Cell survival was analysed by fluorescence activated cell sorting (FACS) and via trypan blue staining. Wildtype and knock out cells were grown until confluency and serum starved for 24 h. FACS was performed on a BD FACSCalibur (BD Biosciences) using Annexin V-FITC and propodium iodide as previously described[1]. Cells were stained with 0.4% TrypanBlue (Thermo Fisher Scientific) and counted in a haemocytometer.

Small RNA sequencing of smEVs

Small RNAs were isolated from control and mutant smEV pellets in triplicate using the Total Exosome RNA and Protein Isolation Kit (4478545, Invitrogen) according to manufacturer's instructions. NGS library prep was performed with NEXTflex Small RNA-Seq Kit V3 following Step A to Step G of Bioo Scientific`s standard protocol (V16.06) using the NEXTFlex 3' SR Adaptor and 5' SR Adaptor (5' rApp /NNNNTGGAATTCTCGGGTGCCAAGG/ 3ddC/ and 5' GUUCAGAGUUCUACAGUCCGACGAUCNNNN, respectively). Libraries were prepared with a starting amount of 24,6ng and amplified in 15 PCR cycles. Amplified libraries were purified by running an 8% TBE gel and size-selected for 18-40nt. Libraries were profiled in a High Sensitivity DNA on a 2100 Bioanalyzer (Agilent technologies) and quantified using the Qubit dsDNA HS Assay Kit, in a Qubit 2.0 Fluorometer (Life technologies). Sequencing was done a NextSeq 500/550 High-throughput Flowcell, SR for 1x 75 cycles plus 7 cycles for the index read.

Small RNA read processing and mapping

The following steps were taken prior to read alignment: (1) adapter trimming with cutadapt v1.9 [REF: 10.14806/ej.17.1.200] (-a TGGAATTCTCGGGTGCCAAGG -O 5 -m 26 -M 45); (2) removal of reads with low-quality calls with fastq_quality_filter (-q 20 -p 100 -Q 33) from the FASTX-Toolkit v0.0.14 [REF: http://hannonlab.cshl.edu/fastx_toolkit]; (3) removal of PCR duplicates making use of the unique molecule identifiers (UMIs) added during library preparation by collapsing reads with the same sequence (including UMIs); (4) Trimming of the UMI sequences (seqtk trimfq -b 4 -e 4) [REF: https://github.com/lh3/setqtk]; and finally (5) removal of reads shorter than 15 nucleotides (seqtk seq-L 15). FastQC was used to asses the quality of reads before and after processing.

miRNA quantification and differential enrichment

miRNA abundance was estimated with mirDeepMApper v.2.0.0.8 (mapper.pl -e -p -h -m -l -j followed by miRDeep2.pl -t mouse -c -d -v). A table of miRNA read counts was prepared using the output of miRdeep2 (miRNA_expressed_all_samples). For miRNAs with multiple entries only the maximal

count was used to avoid redundant entries, and the resulting table of counts used for differential analysis with DESeq2.

miRNA content in EVs

Processed reads were first aligned to the *M. musculus* rRNA sequences to remove contaminating degraded rRNA using bowtie 0.12.8 (bowtie --best -p 4 -v 2 --sam --un). The unmapped reads in this first step, were then mapped to the *M. musculus* genome assembly (gencode, release M10/ GRCm38) with bowtie v0.12.8[2] (bowtie -q --sam --phred33-quals --tryhard --best --strata --chunkmbs 256 -v 1 -M 1). Gene counts were obtained with htseq-count v0.9.0 [anders_htseqpython_2004] (htseq-count-f bam -m intersection-nonempty -s reverse), and the annotation was from Gencode (vM10.chr_patch_hapl_scaff.annotation.gtf).

EV protein identification via mass spectrometry

EV pellets were subjected to a methanol-chloroform precipitation followed by tryptic cleavage as described before[3]. LC-MS/MS analysis was performed on Ultimate3000 RSLCnano systems (Thermo Scientific) coupled to an Orbitrap Fusion Tribrid mass spectrometer (Thermo Scientific) by a nano spray ion source. Tryptic peptide mixtures were injected automatically and loaded at a flow rate of 10 µl/min in 0.1% trifluoroacetic acid in HPLC-grade water onto a nano trap column (Thermo Scientific; Orbitrap Fusion: 2 mm x 10 mm, µPAC Trapping column, 300 nm, 100-200 Å, PharmaFluidics). After 3 min, peptides were eluted and separated on the analytical column (315 µm x 50cm, µPACTM nano-LC columns – 50cm µPACTM C18, 300 nm, 100-200 Å, PharmaFluidics) by a linear gradient from 2% to 30% of buffer B (80% acetonitrile and 0.08% formic acid in HPLC-grade water) in buffer A (2% acetonitrile and 0.1% formic acid in HPLC-grade water) at a flow rate of 300 nl/min over 95 min. Remaining peptides were eluted by a short gradient from 30% to 95% buffer B in 5 minutes. From the high-resolution MS pre-scan with a mass range of 335 to 1500. The Orbitrap Fusion was run in top speed mode with a cycle time of 3 seconds. The normalized collision energy for HCD was set to a value of 30 and the resulting fragments were detected in the ion trap. The lock mass option was activated; the background signal with a mass of 445.12003 was used as lock mass[4]. Every ion selected for fragmentation was excluded for 20 s by dynamic exclusion.

Analysis of mass spectrometry data

MS/MS data were analyzed using the MaxQuant software (version 1.6.1.09; https://maxquant.net/)[5,6]. As a digesting enzyme, Trypsin/P was selected with maximal 2 missed cleavages. Cysteine carbamidomethylation was set for fixed modifications, and oxidation of methionine and N-terminal acetylation were specified as variable modifications. The data were analyzed by label-free quantification with the minimum ratio count of 2. The first search peptide tolerance was set to 20, the main search peptide tolerance to 4.5 ppm and the re-quantify option was selected. For peptide and protein identification, the following subset of the SwissProt database

was used: mouse release 2019_08, #17,027 entries, contaminants were detected using the MaxQuant contaminant search. A minimum peptide number of 2 and a minimum length of 7 amino acids were tolerated. Unique and razor peptides were used for quantification. The match between run options was enabled with a match time window of 0.7 min and an alignment time window of 20 min.

The statistical analysis was done using the Perseus software (version 1.6.2.3; https://maxquant.net/perseus/)[7]. A minimum of 4 biological replicates were used for statistics. Potential contaminants, peptides only identified by side or reverse sequence were removed. Minimum half of the samples must have valid values. Based on the median value, significance A (Benjamini-Hochberg FDR) was calculated. The stability of protein ratios within groups was determined using the student's t-test.

Venn diagrams displaying overlapping proteins inside WT small and large EV populations as well as in WT and mutant small EV preparations were created with Venny 2.1.0 (https://bioinfogp.cnb.csic.es/tools/venny/index.htm). Gene enrichment analysis was performed using GetGo (http://getgo.russelllab.org/)[8]. Top hits for the GO-term "cellular component" and hits identified within the GO-term "biological processes" related to signalling were listed in a table with corresponding GO-term and Fisher-C-PV. Afterwards all proteins were manually screened for function within the Wnt signalling pathway using UniProt (https://www.uniprot.org/). Results were recorded in a separate table.

Quantitative real-time reverse transcriptase polymerase chain reaction

The total RNA was extracted from KM cells treated with siRNA against Anexin1, Alix, Dicer1 or NTC (for list of siRNAs see Supplementary Table 2) using TRIzol reagent (Thermo Fisher, 15596026) following manufacturer's instructions. 1 µg of RNA was reverse transcribed to cDNA using GoScript reverse transcription system (Promega, A5000). cDNA was amplified on a StepOnePlus™ Real-Time PCR System (Applied Biosystems, 4376600) using SYBR Green (Thermo Fisher; Platinum™ SYBR™ Green qPCR SuperMix-UDG, 11733046) according to the manufacturer's recommendation. The following cycling conditions were used: 95 °C for 10 min followed by 40 cycles of 95 for 15 s, 60 for 1 min. Specifcity of the amplifed product was determined by melt curve analysis. Relative target gene expression was normalized to GAPDH and analyzed by comparative Ct or 2ΔΔCT method[9,10]. For a list of primers used, see Supplementary Table 1.

Supplementary Tables

Supplementary Table 1: Information for primers used in study.

Primerlist:

Gene	Species	Forward	Reverse
Anxa1	Mouse	TGATGCAGATGAACTCCGTG	GGCCAGATCTCTTTTCAGCTC
Pdcd6ip	Mouse	TCAAGCAGTGTCAGTACAAGG	TGCGATCTCTTCCCCAAAC
Dicer 1	Mouse	GCTCAGGGAAGACGTTCATC	TTCTGACGGCTGACACTTG
Gapdh	Mouse	CGACTTCAACAGCAACTCCCACTCTTCC	TGGGTGGTCCAGGGTTTCTTACTCCTT

Supplementary Table 2: Information for siRNAs used in study.

Used siRNAs:

Gene	Company	Name	Species	Sequence
Anxa1	IDT	mm.Ri.Anxa1.13.2	Mouse	5'-AUUACGGAAAGUACAGUCAACAUGA-3'<br>3'-CUUAAUUGCCUUUCAUGUCAGUUGUACU-5'
Dicer1	IDT	mm.Ri.Dicer1.13.1	Mouse	5'-GCAGUGAUCAUUCCAAGAUACCGCA-3'<br>3'-UACGUCACUAGUAAGGUUCUAUGGCGU-5'
Dicer1	IDT	mm.Ri.Dicer1.13.3	Mouse	5'-GUACCUGAUAACUAGUUUGAAUCAT-3'<br>3'-UUCAUGGACUAUUGAUCAAACUUAGUA-5'
Pdcd6ip	IDT	mm.Ri.Pdcd6ip.13.3	Mouse	5'- AUGAGAGAAGCUACGACUUUGGCAA -3'<br>3'-UCUACUCUCUUCGAUGCUGAAACCGUU-5'

Supplementary Data Legends

Supplementary Data 1. Proteins identified in smEVs Serum Starved.

sEV protein cargo identified via liquid chromatography mass spectrometry. smEVs were harvested from WT, Bbs4 and Bbs6 KM cells post serum starvation.

Supplementary Data 2. Proteins identified in smEVs grown with Serum.

sEV protein cargo identified via liquid chromatography mass spectrometry. smEVs were harvested from WT, Bbs4 and Bbs6 KM cells grown in the presence of serum.

Supplementary Data 3. Proteins identified in lgEVs Serum Starved.

lgEV protein cargo identified via liquid chromatography mass spectrometry. lgEVs were harvested from WT KM cells post serum starvation.

Supplementary Data 4. RNA biotypes identified in smEVs Serum Starved.

Mean percentage biotypes per library identified via small RNA sequencing of sEV cargo harvested from WT, Bbs4 and Bbs6 KM cells post serum starvation.

Supplementary Data 5. miRNAs identified in smEVs Serum Starved.

sEV protein cargo identified via small RNA sequencing. smEVs were harvested from WT, Bbs4 and Bbs6 KM cells post serum starvation.

Supplementary References

1. Patnaik, S. R. *et al.* Bardet–Biedl Syndrome proteins regulate cilia disassembly during tissue maturation. *Cell. Mol. Life Sci.* **76**, 757–775 (2019).
2. Langmead, B., Trapnell, C., Pop, M. & Salzberg, S. L. Ultrafast and memory-efficient alignment of short DNA sequences to the human genome. *Genome Biol.* (2009). doi:10.1186/gb-2009-10-3-r25
3. Gloeckner, C. J., Boldt, K. & Ueffing, M. Strep/FLAG tandem affinity purification (SF-TAP) to study protein interactions. *Current Protocols in Protein Science* (2009). doi:10.1002/0471140864.ps1920s57
4. Olsen, J. V. *et al.* Parts per million mass accuracy on an orbitrap mass spectrometer via lock mass injection into a C-trap. *Mol. Cell. Proteomics* (2005). doi:10.1074/mcp.T500030-MCP200
5. Cox, J. & Mann, M. MaxQuant enables high peptide identification rates, individualized p.p.b.-range mass accuracies and proteome-wide protein quantification. *Nat. Biotechnol.* (2008). doi:10.1038/nbt.1511
6. Cox, J. *et al.* A practical guide to the maxquant computational platform for silac-based quantitative proteomics. *Nat. Protoc.* (2009). doi:10.1038/nprot.2009.36
7. Tyanova, S. *et al.* The Perseus computational platform for comprehensive analysis of (prote)omics data. *Nature Methods* (2016). doi:10.1038/nmeth.3901
8. Boldt, K. *et al.* An organelle-specific protein landscape identifies novel diseases and molecular mechanisms. *Nat. Commun.* (2016). doi:10.1038/ncomms11491
9. Pfaffl, M. W. A new mathematical model for relative quantification in real-time RT–PCR. *Nucleic Acids Res.* **29**, e45–e45 (2001).
10. KJ, L. & TD, S. Analysis of relative gene expression data using real-time quantitative PCR and the 2(-Delta Delta C(T)) Method. *Methods* **25**, 402–408 (2001).

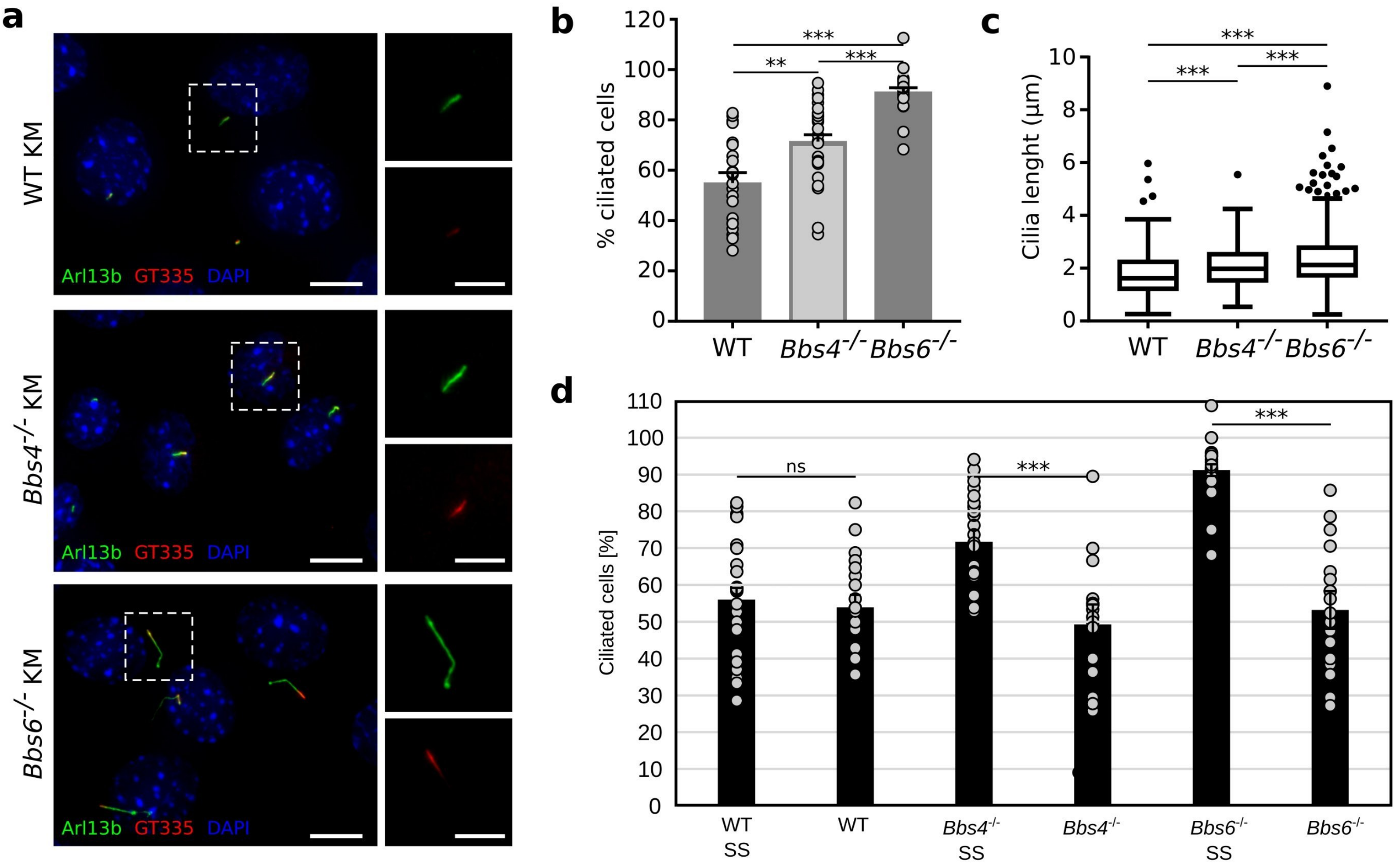

Supp. Fig.1: Cilia number and length in control and cilia mutant KM cells. (a) Representative images of control and cilia mutant cells stained with Arl13b (cilia membrane), GT335 (basal body & transition zone) and DAPI (nucleus) (Scale bar: 10µm). Close up images show one Arl13b and GT335 positive cilium (Scale bar: 5µm). (b) Quantification of number of ciliated cells showed an increase of ciliation inside the cilia mutant cells (NWT=558 cells; NBbs4-/-=453 cells; NBbs6-/-=537 cells). (c) Bbs4-/- and Bbs6-/- cells display significantly longer cilia than control cells (pWT vs. Bbs4-/-=6.8E-7; pWT vs. Bbs6-/-=2.54E-15; pBbs4-/- vs. Bbs6-/-=0.002). (d) Comparison of cilia number of serum starved and serum-fed KM cells showed a decrease in cilia number upon serum addition in cilia mutant cells but not in control cells. (unpaired t-Test pWT=0.886; pBbs4-/-=0.00004; Mann-Whitney-U-Test pBbs6-/-=1.45E-7). (b,d) Data are presented as mean values +/- SEM . (c) Boxplots show median, interquartile range, and maximum and minimum within 1.5 interquartile range. (b,c,d) n.s., p > 0.05, *p ≤ 0.05, **p<0.01, ***p<0.001. Source data are provided as a Source Data file.

Supplemetary Figure 1

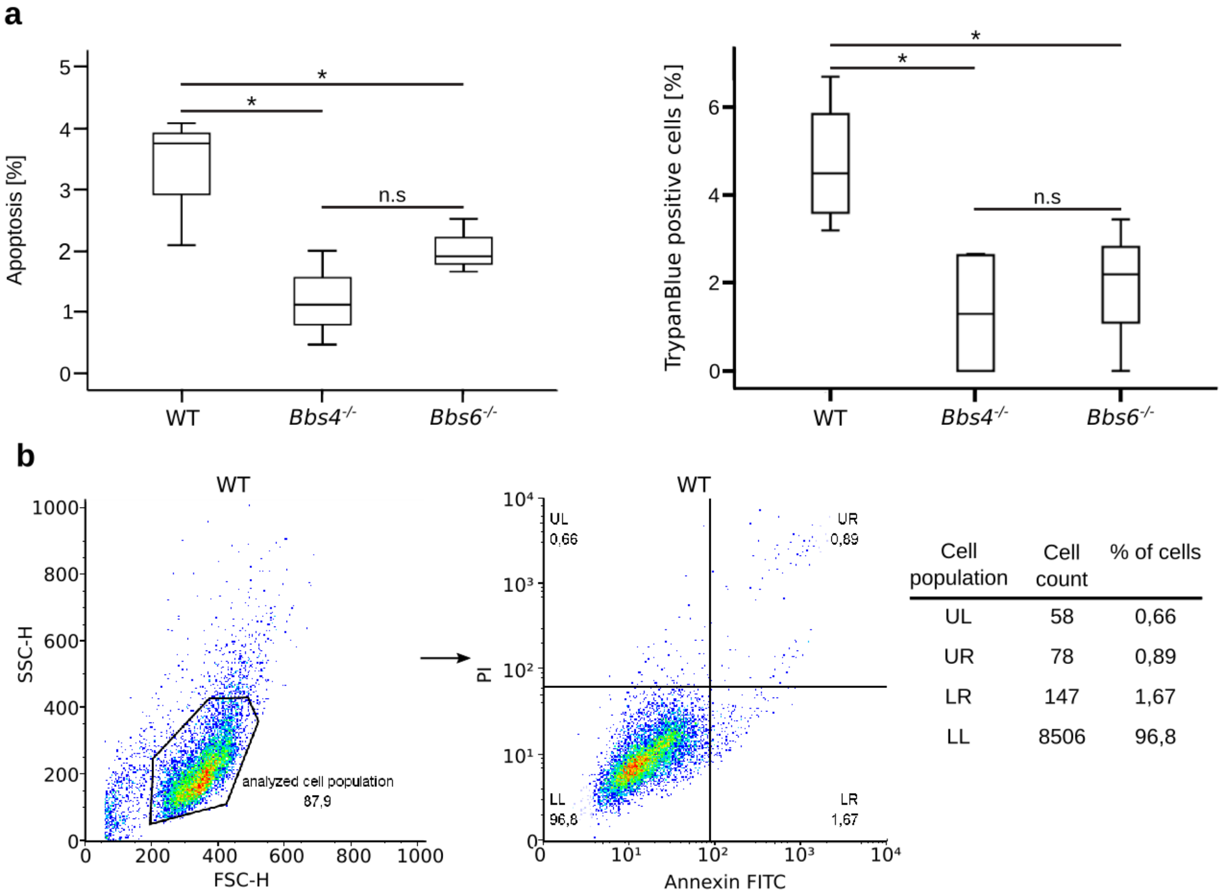

Cell population	Cell count	% of cells
UL	58	0,66
UR	78	0,89
LR	147	1,67
LL	8506	96,8

Supp. Fig. 2: Apoptosis rate in control and cilia mutant KM cells. (a) Quantification of the number of dead cells in cultured control and cilia mutant KM cells using fluorescence-activated cell sorting (FACS) and Trypan Blue staining revealed less cell death in the cilia mutant samples compared to control (Mann-Whitney-U Test). Overall, the apoptosis rate of all three cell lines was low (1-6%). Boxplots show median, interquartile range, and maximum and minimum within 1.5 interquartile range. n.s., $p > 0.05$, $*p \leq 0.05$, $**p<0.01$, $***p<0.001$. Source data are provided as a Source Data file. (b) Representative FACS analysis. Cells were gated based on size and granularity (forward and side scatter). The highest proportion of cells in the lower left (LL) square, representing the living cell population. Apoptotic cells were displayed in the lower right (LR) square.

Supplemetary Figure 2

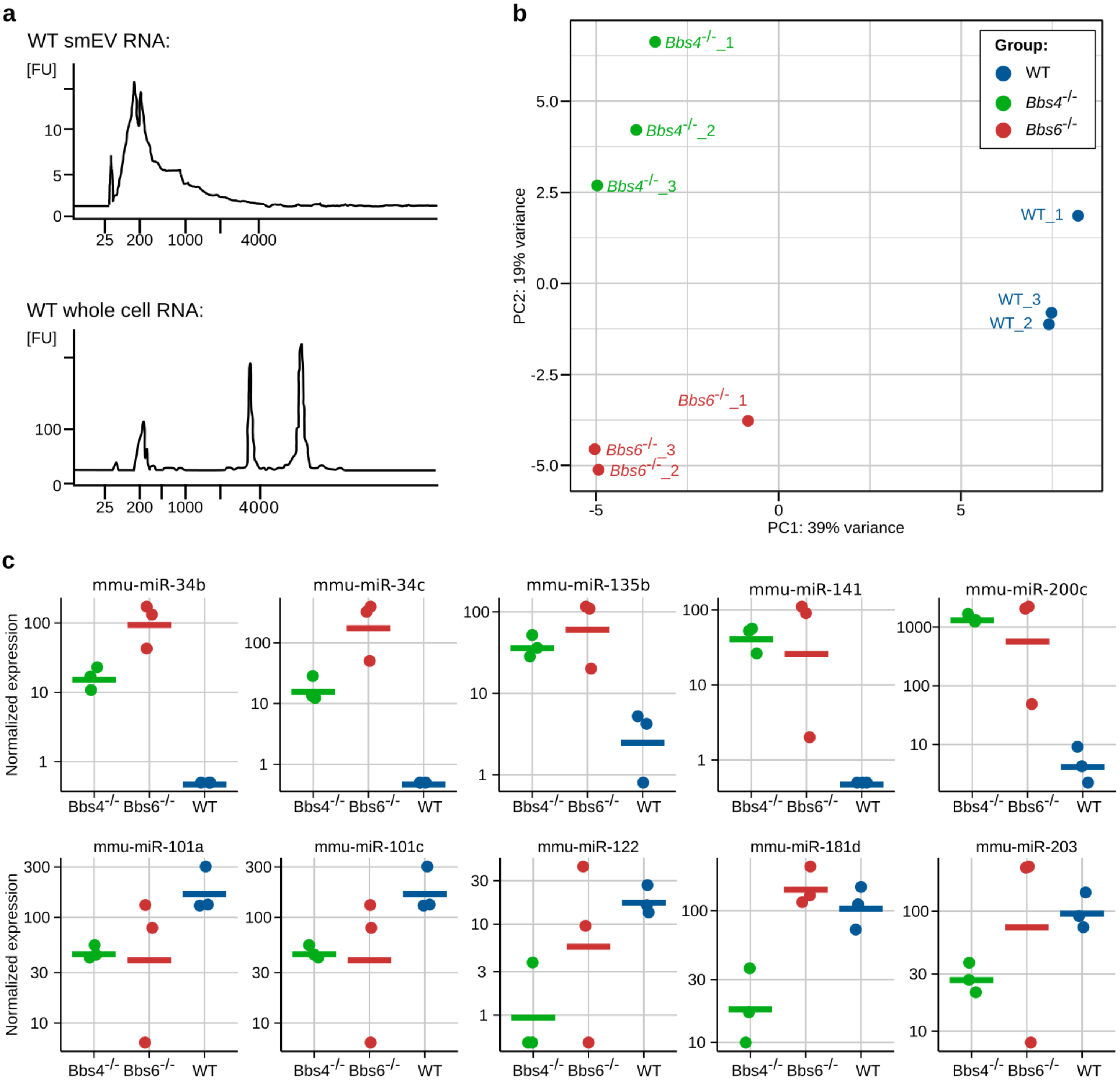

Supp.Fig. 4: miRNAs loaded into smEVs released from cilia mutant cells. (a) RNA profiles from control whole cells vs control smEVs showed an enrichment of small RNAs ranging between 25nm – 1000nm in smEVs. (b) Principal component analysis of miRNA sequencing from triplicate sample preparation. Triplicates from each genotype clustered together but where distinctly separate from different genotypes. (c) Individual dot plots of differentially regulated miRNA. Plotted are the expression levels of distinct miRNAs in each separate preparation (green=Bbs4-/-; red=Bbs6-/- & blue=WT).

Supplemetary Figure 4

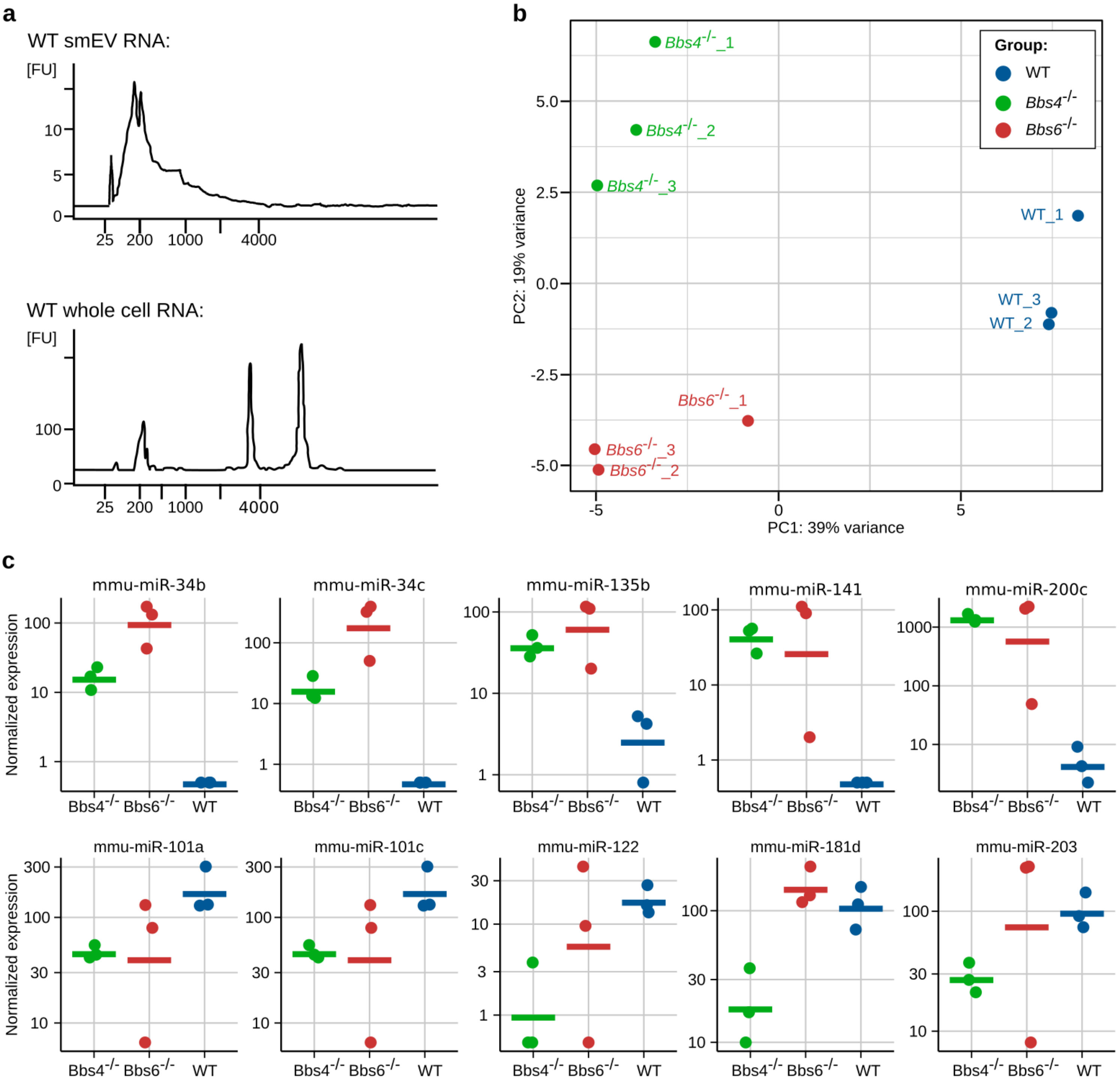

Supp.Fig. 4: miRNAs loaded into smEVs released from cilia mutant cells. (a) RNA profiles from control whole cells vs control smEVs showed an enrichment of small RNAs ranging between 25nm – 1000nm in smEVs. (b) Principal component analysis of miRNA sequencing from triplicate sample preparation. Triplicates from each genotype clustered together but where distinctly separate from different genotypes. (c) Individual dot plots of differentially regulated miRNA. Plotted are the expression levels of distinct miRNAs in each separate preparation (green=Bbs4-/-; red=Bbs6-/- & blue=WT).

Supplemetary Figure 4

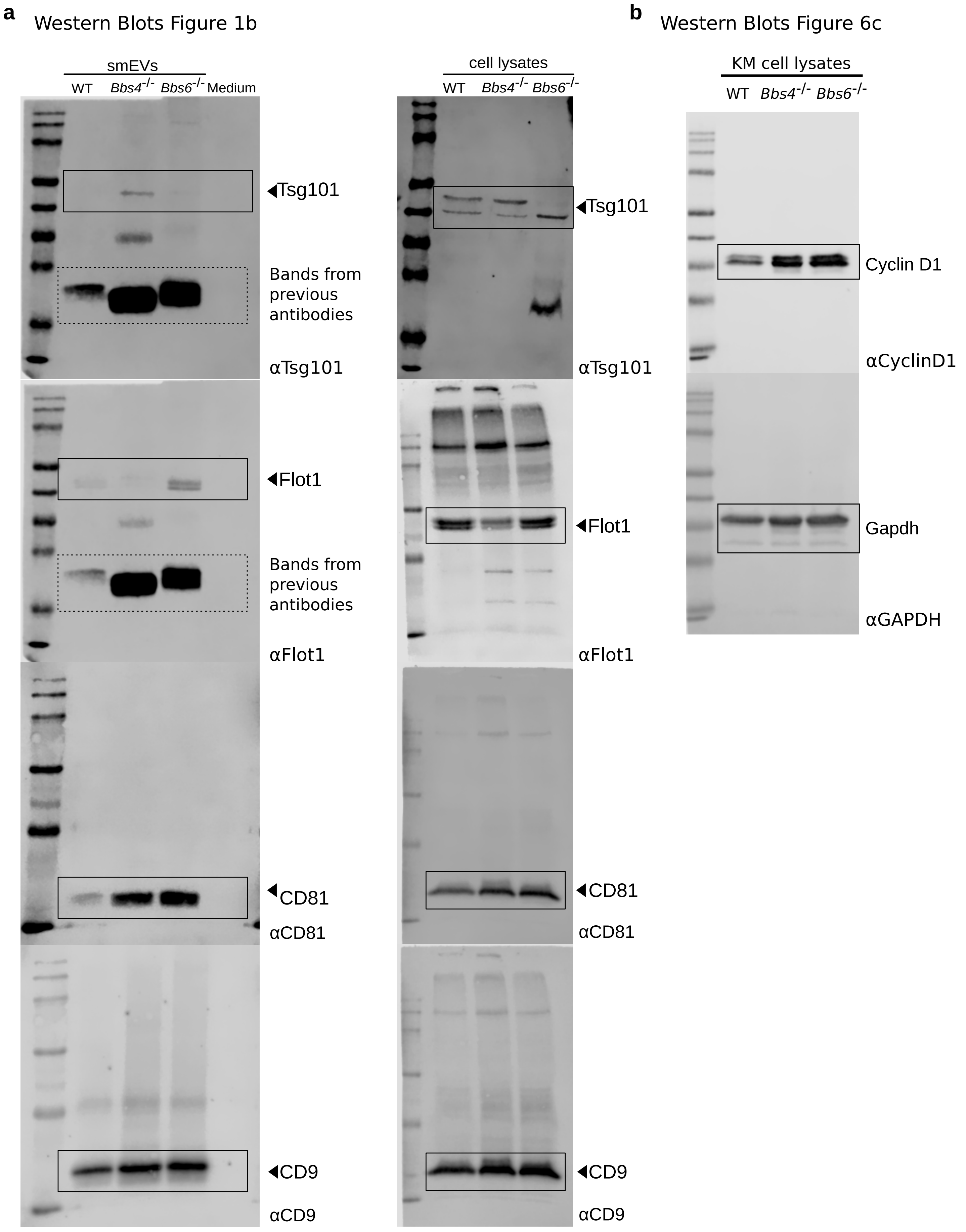

Supp.Fig. 5: Original Western Blots. (a) Original uncropped Western blots shown in Figure 1b. (b) Original uncropped Western blots shown in Figure 6c.

Supplementary Figure 5
