# Supplementary material for: Bardet-Biedl syndrome proteins modulate the release of bioactive extracellular vesicles
Source: Nat Commun. 2021 Sep 27;12:5671. doi: 10.1038/s41467-021-25929-1 (PMC8476602; doi:10.1038/s41467-021-25929-1)
Supplement: Supplementary file 8 — Description of additional supplementary files [file 41467_2021_25929_MOESM8_ESM.docx]

Description of additional supplementary files

Title: Supplementary Data 1.

Description :Proteins identified in smEVs Serum Starved. sEV protein cargo identified via liquid chromatography mass spectrometry. smEVs were harvested from WT, Bbs4 and Bbs6 KM cells post serum starvation.

Title: Supplementary Data 2.

Description: Proteins identified in smEVs grown with Serum. sEV protein cargo identified via liquid chromatography mass spectrometry. smEVs were harvested from WT, Bbs4 and Bbs6 KM cells grown in the presence of serum.

Title: Supplementary Data 3.

Description: Proteins identified in lgEVs Serum Starved. lgEV protein cargo identified via liquid chromatography mass spectrometry. lgEVs were harvested from WT KM cells post serum starvation.

Title: Supplementary Data 4.

Description: RNA biotypes identified in smEVs Serum Starved. Mean percentage biotypes per library identified via small RNA sequencing of sEV cargo harvested from WT, Bbs4 and Bbs6 KM cells post serum starvation.

Title: Supplementary Data 5.

Description: miRNAs identified in smEVs Serum Starved. sEV protein cargo identified via small RNA sequencing. smEVs were harvested from WT, Bbs4 and Bbs6 KM cells post serum starvation.
