# Supplementary material for: Bardet-Biedl syndrome proteins modulate the release of bioactive extracellular vesicles
Source: Nat Commun. 2021 Sep 27;12:5671. doi: 10.1038/s41467-021-25929-1 (PMC8476602; doi:10.1038/s41467-021-25929-1)
Supplement: Supplementary file 9 — Reporting Summary [file 41467_2021_25929_MOESM9_ESM.pdf]

nature research

Corresponding author(s): Helen May-Simera

Last updated by author(s): Jul 22, 2021

# Reporting Summary

Nature Research wishes to improve the reproducibility of the work that we publish. This form provides structure for consistency and transparency in reporting. For further information on Nature Research policies, see our Editorial Policies and the Editorial Policy Checklist.

## Statistics

For all statistical analyses, confirm that the following items are present in the figure legend, table legend, main text, or Methods section.

| n/a | Confirmed | |
|---|---|---|
| ☐ | ☒ | The exact sample size (*n*) for each experimental group/condition, given as a discrete number and unit of measurement |
| ☐ | ☒ | A statement on whether measurements were taken from distinct samples or whether the same sample was measured repeatedly |
| ☐ | ☒ | The statistical test(s) used AND whether they are one- or two-sided<br>*Only common tests should be described solely by name; describe more complex techniques in the Methods section.* |
| ☒ | ☐ | A description of all covariates tested |
| ☐ | ☒ | A description of any assumptions or corrections, such as tests of normality and adjustment for multiple comparisons |
| ☐ | ☒ | A full description of the statistical parameters including central tendency (e.g. means) or other basic estimates (e.g. regression coefficient) AND variation (e.g. standard deviation) or associated estimates of uncertainty (e.g. confidence intervals) |
| ☐ | ☒ | For null hypothesis testing, the test statistic (e.g. *F*, *t*, *r*) with confidence intervals, effect sizes, degrees of freedom and *P* value noted<br>*Give P values as exact values whenever suitable.* |
| ☒ | ☐ | For Bayesian analysis, information on the choice of priors and Markov chain Monte Carlo settings |
| ☒ | ☐ | For hierarchical and complex designs, identification of the appropriate level for tests and full reporting of outcomes |
| ☒ | ☐ | Estimates of effect sizes (e.g. Cohen's *d*, Pearson's *r*), indicating how they were calculated |

*Our web collection on statistics for biologists contains articles on many of the points above.*

## Software and code

Policy information about availability of computer code

| | |
|---|---|
| Data collection | LAS AF Lite (Version 2.3.5 Build 5379; BlindDeblur Algorithm, one iteration step; Leica Microsystems CMS GmbH, Wetzlar)<br>Nanosight 2.3 software (Malvern, Herrenberg, Germany)<br>Image Studio Version 5.2 software (LI-COR Bioscience)<br>V_3.23_12/10_Infinite (Tecan Trading AG, Switzerland) |
| Data analysis | MaxQuant (https://maxquant.net/; version 1.6.1.09)<br>Perseus (version 1.6.2.3; https://maxquant.net/perseus/)<br>SPSS Statistics 23.0 (IBM, USA).<br>GetGO:Gene enrichment analysis tool (http://getgo.russelllab.org/)<br>Fiji/Image J (Version 1.50 a; Wayne Rasband NIH)<br>miRDB (http://mirdb.org/)<br>TargetScanMouse (http://www.targetscan.org/mmu_71/)<br>Cell Quest Pro 6.0 (BD Biosciences) |

For manuscripts utilizing custom algorithms or software that are central to the research but not yet described in published literature, software must be made available to editors and reviewers. We strongly encourage code deposition in a community repository (e.g. GitHub). See the Nature Research guidelines for submitting code & software for further information.

## Data

Policy information about availability of data

All manuscripts must include a data availability statement. This statement should provide the following information, where applicable:

- Accession codes, unique identifiers, or web links for publicly available datasets
- A list of figures that have associated raw data
- A description of any restrictions on data availability

The authors declare that the data supporting the findings of this study are available within the paper and its supplementary information. Deep-sequencing data generated in this study have been deposited to the NCBI GEO and are available under accession number GEO: GSE153227. https://www.ncbi.nlm.nih.gov/geo/query/acc.cgi?acc=GSE153227.Mass spectrometry proteomics data has been submitted to ProteomeXchange via the PRIDE database. Project accession: PXD020466. https://www.ebi.ac.uk/pride/archive/projects/PXD020466

# Field-specific reporting

Please select the one below that is the best fit for your research. If you are not sure, read the appropriate sections before making your selection.

☒ Life sciences ☐ Behavioural & social sciences ☐ Ecological, evolutionary & environmental sciences

For a reference copy of the document with all sections, see nature.com/documents/nr-reporting-summary-flat.pdf

# Life sciences study design

All studies must disclose on these points even when the disclosure is negative.

| | |
|---|---|
| Sample size | No sample size calculation was performed. The exact sample size is stated in the methods section and in the corresponding figure legends. The sample size was chosen equal or more than 3 to run a student's t-test or other statistics analysis. We performed for every experiment at least three biological independent experiments. |
| Data exclusions | Cells has to be genotyped afer thawing. NTA data after knock down experiments were excluded when knock down did not work. This was confirmed via qPCR. |
| Replication | Experiments were replicated several times (2-5) with reproducible results, as indicated in each figure legend. |
| Randomization | Not relevant. Experiments were performed on mutant versus control cells. |
| Blinding | During RNA library preparation and miRNA sequencing samples were blinded of genotypes. In microscopy analysis, the same settings were used in data collection and data analysis. IIn other experiments blinding was not possible as sample preparation were performed by the same person. |

# Reporting for specific materials, systems and methods

We require information from authors about some types of materials, experimental systems and methods used in many studies. Here, indicate whether each material, system or method listed is relevant to your study. If you are not sure if a list item applies to your research, read the appropriate section before selecting a response.

### Materials & experimental systems

| n/a | Involved in the study | |
|---|---|---|
| ☐ | ☒ | Antibodies |
| ☐ | ☒ | Eukaryotic cell lines |
| ☒ | ☐ | Palaeontology and archaeology |
| ☒ | ☐ | Animals and other organisms |
| ☐ | ☒ | Human research participants |
| ☒ | ☐ | Clinical data |
| ☒ | ☐ | Dual use research of concern |

### Methods

| n/a | Involved in the study | |
|---|---|---|
| ☒ | ☐ | ChIP-seq |
| ☐ | ☒ | Flow cytometry |
| ☒ | ☐ | MRI-based neuroimaging |

## Antibodies

| | |
|---|---|
| Antibodies used | CD9 (1:1000, clone KMC8, #553758, BD Pharmingen™), CD81 (1:1000, clone B-11, sc-166029, Santa Cruz), Flotillin-1 (1:1000, #F1180, Sigma-Aldrich), Tsg101 (1:1000, clone 4A10, GeneTex Irvine), Cyclin D1 (1:1000, #55506, Cell Signaling), Arl13b (1:800, #17711-1-AP, Proteintech); GT335 (1:200, #AG-20B-0020-C100, Adipogen Life Sciences); Gapdh (1:1000, 6004-1-lg, Proteintech) TRITC-Rhodamine-Phalloidin (R415; Thermo Fisher Scientific); anti-mouse igG Alexa Flour555 (A-31570, Thermo Fisher Scientific); anti-rabbit igG Alexa Flour 488 (A-11034, Thermo Fisher Scientific); DAPI (#6843; Carl Roth); IRDye® 680RD anti-Rabbit IgG (#925-68073, Li-COR |

| | |
|---|---|
| | Bioscience); IRDye® 680RD anti-Mouse IgG (#925-68072, IRDye® 800CW anti-Mouse IgG (#925-32212, Li-COR Bioscience); RDye® 800CW anti-Rabbit IgG (#926-32213, Li-COR Bioscience); Goat anti-rat IgG (#SA5-10278, Thermo Fisher Scientific) |
| Validation | All the antibodies used in this study were commercial antibodies and were only used for applications, with validation procedures described on the following sites of the manufacturers:<br>CD9: https://www.bdbiosciences.com/en-us/products/reagents/flow-cytometry-reagents/research-reagents/single-color-antibodies-ruo/purified-na-le-rat-anti-mouse-cd9.553758<br>CD81: https://www.scbt.com/de/p/cd81-antibody-b-11<br>Flotillin-1: https://www.sigmaaldrich.com/DE/de/product/sigma/f1180#<br>Tsg101: https://www.genetex.com/Product/Detail/TSG101-antibody-4A10/GTX70255<br>CyclinD1: https://www.cellsignal.com/products/primary-antibodies/cyclin-d1-e3p5s-xp-rabbit-mab/55506<br>Arl13b: https://www.ptglab.com/products/ARL13B-Antibody-17711-1-AP.htm<br>GT335: https://adipogen.com/ag-20b-0020-anti-polyglutamylation-modification-mab-gt335.html/<br>Gapdh: https://www.ptglab.com/products/GAPDH-Antibody-60004-1-Ig.htm |

## Eukaryotic cell lines

Policy information about cell lines

| | |
|---|---|
| Cell line source(s) | Kidney Medullar Cells: Immortalized primary culture from mutant mouse lines from P. Beales (Institute of Child Health, UCL, London UK);<br>UREC cultures Generated from Patient Urine;<br>TCF/LEF Reporter-HEK293 cell line (BPS Bioscience, San Diego, CA, USA)<br>HEK293T cells (ATCC®, Teddington) |
| Authentication | Hernandez-Hernandez, V. et al. Bardet-biedl syndrome proteins control the cilia length through regulation of actin polymerization. Hum. Mol. Genet. 22, 3858–3868 (2013). |
| Mycoplasma contamination | Not tested |
| Commonly misidentified lines<br>(See ICLAC register) | No commonly misidentified cell lines were used in this study. |

## Human research participants

Policy information about studies involving human research participants

| | |
|---|---|
| Population characteristics | We generated Urine Derived Epithelial Cultures from Urine collected from a Bardet Biedl Syndrome patient and age and gender matched control. |
| Recruitment | The patient was recruited via the German Bardet Biedl Syndrome Patient society a subgroup of the Pro Retina Society. Selection was based on genotype and geographic location. Age and gender match was chosen in line with the selected patient. |
| Ethics oversight | Urine was collected with informed consent and ethical approval (Landesärztekammer Rheinland-Pfalz 2019-14118). |

Note that full information on the approval of the study protocol must also be provided in the manuscript.

## Flow Cytometry

### Plots

Confirm that:

☒ The axis labels state the marker and fluorochrome used (e.g. CD4-FITC).

☒ The axis scales are clearly visible. Include numbers along axes only for bottom left plot of group (a 'group' is an analysis of identical markers).

☒ All plots are contour plots with outliers or pseudocolor plots.

☒ A numerical value for number of cells or percentage (with statistics) is provided.

### Methodology

| | |
|---|---|
| Sample preparation | Wildtype and knock out mouse kidney medullary cells. Samples were centrifuged at 800 g for 3 minutes and the pellet were diluted in RNase-free PBS. |
| Instrument | BD FACS Calibur |
| Software | Cell Quest Pro. 6.0; Macintosh System Software 10.6.8 |
| Cell population abundance | Cell populations were generated from WT and mutant mice. These were genotyped to confirm that a pure cell population was maintained. |

Gating strategy

Cells were gated based on size and granularity (forward and side scatter).

☒ Tick this box to confirm that a figure exemplifying the gating strategy is provided in the Supplementary Information.
